# Collective Motion and Self-Organization of a Swarm of UAVs: A Cluster-Based Architecture

**DOI:** 10.3390/s21113820

**Published:** 2021-05-31

**Authors:** Zain Anwar Ali, Zhangang Han, Rana Javed Masood

**Affiliations:** 1 School of Systems Science, Beijing Normal University, Zhuhai 519085, China; 2 Department of Electrical Engineering, Usman Institute of Technology, Karachi 75300, Pakistan; rjmasood@uit.edu

**Keywords:** particle swarm optimization, multi-agent system, vicsek model

## Abstract

This study proposes a collective motion and self-organization control of a swarm of 10 UAVs, which are divided into two clusters of five agents each. A cluster is a group of UAVs in a dedicated area and multiple clusters make a swarm. This paper designs the 3D model of the whole environment by applying graph theory. To address the aforesaid issues, this paper designs a hybrid meta-heuristic algorithm by merging the particle swarm optimization (PSO) with the multi-agent system (MAS). First, PSO only provides the best agents of a cluster. Afterward, MAS helps to assign the best agent as the leader of the *n*th cluster. Moreover, the leader can find the optimal path for each cluster. Initially, each cluster contains agents at random positions. Later, the clusters form a formation by implementing PSO with the MAS model. This helps in coordinating the agents inside the *n*th cluster. However, when two clusters combine and make a swarm in a dynamic environment, MAS alone is not able to fill the communication gap of *n* clusters. This study does it by applying the Vicsek-based MAS connectivity and synchronization model along with dynamic leader selection ability. Moreover, this research uses a B-spline curve based on simple waypoint defined graph theory to create the flying formations of each cluster and the swarm. Lastly, this article compares the designed algorithm with the NSGA-II model to show that the proposed model has better convergence and durability, both in the individual clusters and inside the greater swarm.

## 1. Introduction

In the last decade, research on unmanned aerial vehicles (UAV) has enormously increased due to its awareness with the help of modern social media. On one hand, at the initial level, researchers are taking interest in modeling and controlling a single aerial vehicle. On the other hand, more and more academics are now studying multi-UAV-based scenarios i.e., trajectory tracking, formation control, and path planning of swarms [1,2,3]. Scientists are studying the natural behavior of a flock of birds, how the birds do the tasks and successfully cooperate within a flock. Researchers in [4,5,6] took the initiative to design artificial intelligence-based bio-inspired algorithms, i.e., ant colony optimization (ACO), particle swarm optimization (PSO), and pigeon-inspired optimization (PIO) to control robots in formation or swarm.

*Motivation:* The main motivation behind this research is to use the insights gained from the natural behavior of birds and ants and apply them to robots. There are multiple studies available that apply these kinds of bio-inspired algorithms to different kinds of aerial and ground robots [7,8,9]. Combining a bio-inspired algorithm with a multi-agent system is also a popular idea among researchers nowadays. This study intends to contribute to this growing research area and apply the aforementioned hybrid algorithm to two clusters of UAVs that turn into one big swarm.

*Related Work:* There are multiple research studies concerning the collective motion of a swarm of UAVs and using different approaches to analyze them. In [10], the researchers used a group of UAVs for fighting forest fires. The researchers in [10] used PSO to help the UAVs plot the most optimal paths toward the fire. In [11], academics applied the PSO for the path planning of a team of UAVs used for 3D surveillance. Teng, H. et al. [11] also presented a fitness function with multiple objectives, including power consumption, flight risk, and area priority. Another study [12] used PSO to plan paths for the formation of multiple UAVs. In [13], researchers used PSO for the trajectory planning of a team of autonomous UAVs. However, classic PSO can get stuck in the local optimum and is slow to converge. This study proposes to use MAS in conjunction with PSO to help reduce the aforementioned issues.

MAS consists of agents that are autonomous and these agents update their position relative to their neighbors’ position. MAS has wide-ranging applications from micro-grid control [14] to tracking control of quad-rotor [15]. In [16], a multi-agent system is used to control the formation of multiple UAVs. In [17], researchers used MAS to transport different loads using a group of aircrafts. One study [18] also discussed applications of MAS to fight the COVID-19 pandemic. Mostly, an external entity drives the agents to the target. MAS designates some of the agents as leaders and the rest of the agents start to follow them. Using this method, big swarms can be controlled using few leaders with the help of MAS.

Recently, some studies have explored the hybrid strategy of applying PSO with MAS. In [19], researchers used the combinations of PSO and MAS to optimize the photovoltaic (PV) systems and battery energy storage systems. In [20], researchers treated PSO as a kind of multi-agent system and used this approach for allocating tasks to subgroups within the bigger structure. Biswas, S. et al. [21] presented a hybrid PSO and MAS approach for the path planning and obstacle avoidance of both dynamic and static threats. Similarly, academics proposed a method to defend against distributed denial-of-service (DDoS) attacks using MAS and the agents used the PSO to communicate [22, 23].

In [24], Vicsek et al. presented a multi-agent model now known as the Vicsek model. It consists of *N* autonomous agents, which have absolute velocities in discrete time. These agents follow the direction of their immediate neighbors. Vicsek et al. [24] concluded that all agents will move in the same direction given their density is large and the external noise is small. This phenomenon is known as synchronization. Recently, scientists have used the Vicsek model for a variety of applications. In [25], researchers used the adaptive Vicsek model to reduce the external disturbances and noise, and retain the formation in a hazardous environment.

Multiple studies use bio-inspired algorithms in multi-agent systems. Leitão, P. et al. [26] used a bio-inspired multi-agent system for enhancing the efficiency of manufacturing systems and industrial automation. Another research [27] showed how a nature-inspired multi-agent system can be effectively used to model pervasive computing systems. In [28], researchers designed a decentralized autonomous robot navigation controller for multi-agent systems. This bio-inspired navigation controller can be used to control a set of robots and allow them to reach the desired location without colliding with static or moving obstacles. In another study [29], researchers presented a bio-inspired multi-agent system for precision agriculture and joint survey missions.

*Contributions:* This research work deals with the self-organization, collective motion, control of a swarm of 10 UAVs, and their definite formation. The following are the major contributions of this research;

➢ Proposing a hybrid meta-heuristic algorithm by merging the particle swarm optimization (PSO) with the multi-agent system (MAS) along with the dynamic leader selection.
➢ Designing an algorithm that ensures fast convergence, synchronization, and connectivity between the agents of both clusters.
➢ Resolving the issues of self-synchronization and collective motion of a swarm of 10 UAVs.
➢ Validating the performance of the proposed algorithm by using real-time-based numerical simulation, which demonstrates the effectiveness of hierarchical architecture.

*Organization:* The structure of the study is as follows: Section 2 defines the problem formulation and statement of issues as well as the implementation of the hierarchal-based solution framework. Section 3 demonstrates the preliminaries and a complete system model of architecture. It contains the basic concept of terrain environment, allocation of clusters, and the system model. Moreover, Section 4 has the overall design approach of the proposed strategy including the PSO, Vicsek-based MAS model, its synchronization & connectivity, and dynamic leader selection. Section 5 presents the flowchart and the algorithm of the proposed method. Section 6 offers computer-based simulations and discusses their results. Finally, Section 7 concludes the overall article.

## 2. Problem Description and Solution Architecture

This section presents the issue of the collective motion and self-organization model of a swarm of 10 UAVs. In addition, this section also presents our cluster-based hierarchical solution architecture to solve these issues.

### 2.1. Problem Statements

To accomplish a complicated scenario or task, a group of fixed-wing UAVs sometimes break up into several distinct clusters. Each of the clusters is responsible to perform subtasks. Afterward, when the individual clusters fulfill the desired sub-task, they will merge into a common cluster and start patrolling using the collective motion and self-organizing behavior according to the requirement of the mission. Hence, the formation of the swarms of UAVs can vary over time during the flight. The entire mission is further divided into two scenarios.

**Figure 1:**
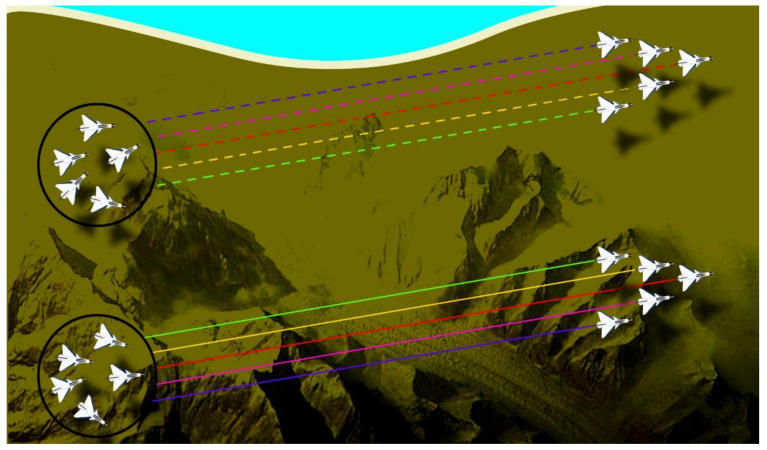
Scenario 1 containing 10 UAVs divided into two clusters in random positions on the left; the two clusters are in leader-follower formation on the right.

*Problem Statement I:* Figure 1 presents the first scenario. Ten fixed-wing UAVs are divided into two clusters and each cluster has a leader and four followers. It means five UAVs in each cluster collaboratively can perform the subtask. The terrain model contains different obstacles like mountains. Aerial vehicles have a challenging time avoiding the hazardous peaks, maintain their strategy, and reach at the designated area safely. Hence, it is necessary to maintain the desired synchronization and connectivity with their neighbor agents. It is also essential to maintain the leader-follower formation when the UAVs pass through narrow gaps.

**Figure 2:**
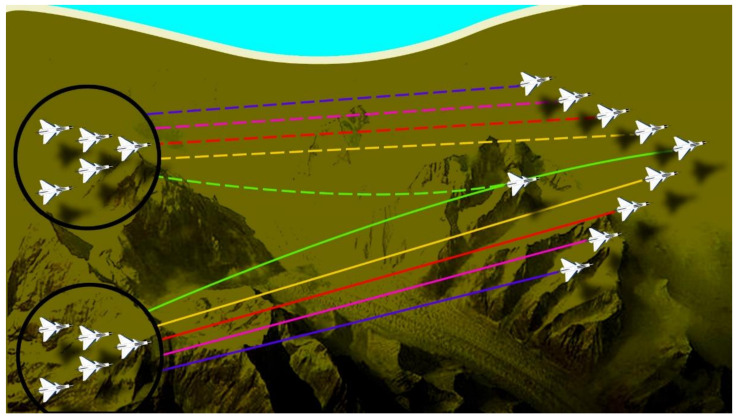
Scenario 2 containing 10 UAVs divided into two clusters in leader-follower formation on the left, the two clusters merge into one swarm on the right.

*Problem Statement II:*Figure 2 presents the second scenario. Scenario 2 starts after successfully maintaining the formation of both the clusters and fulfilling their separate mission tasks in scenario 1. Afterward, both the clusters join in a common cluster called a swarm. At that instant of time, perfect coordination or communication between both the clusters is required to choose which leader will lead the formation of a swarm now. To help achieve the above objective, the algorithm will dynamically select the leader.

### 2.2. Solution Architecture

**Figure 3:**
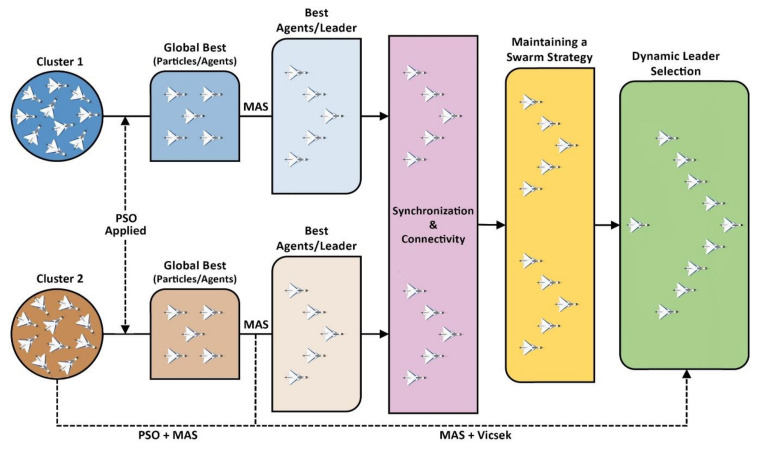
The architecture of the proposed solution.

Figure 3 illustrates the solution architecture of this study. It is evident from the figure that, at first, UAVs were not organized and were in random positions in the cluster. After applying the PSO algorithm in the first phase, the UAVs structure themselves into a strict formation. PSO also determines the best particle of each cluster. In the next step, MAS designates the best agent as the leader of that cluster and the rest of the agents start to follow the leader. Now, in the second phase, Vicsek MAS ensures the synchronization and connectivity between two clusters and also between individual agents within the clusters. Afterward, the two clusters merge to form a swarm and the algorithm dynamically selects the leader of the swarm according to mission requirements.

## 3. Preliminaries and System Model

This section defines the terrain environment and the allocation or division of clusters with the help of graph theory. Next, this section will define the system model, which is followed by the swarm 3D model.

### 3.1. Terrain Environment

In this study, the 3D landscape used to develop the whole scenario can be given as [12];

(a) $$z\left(x,y\right)=\sin\left(y+d\right)+e\times \sin\left(x\right)+f\times \cos\left(g\sqrt{x^{2}+y^{2}}\right)+h\times \cos\left(x\right)+i\times \sin\left(j\sqrt{x^{2}+y^{2}}\right)+k\times \cos\left(y\right)$$

In the above equation, (*d*, *e*, *f*, *g*, *h*, *i*, *j*, and *k*) are all constants and their values are determined according to the requirement of the terrain. For example, these constants determine the number of peaks in a simulated environment, their heights, and the space between these peaks. The (*x*, *y*) is the horizontal position and *z* is the height of its vertical plane.

### 3.2. Allocation of Cluster

This subsection defines the classical graph theory terminologies and their notations and how they are used in the allocation of clusters [30,31]. An undirected graph is defined by Ĝ=〈V,E〉. This graph contains V, which is the vertex set, and E, which is the edge set. These sets are used to define the whole hierarchical architecture and have all the information about the start or takeoff points of UAVs in $U_{T}$. $U_{T}$ is a set of UAVs in a cluster and can be defined as $U_{T}=\left(1,2,\ldots ,\;N\right)$. Whereas the terrain cost is defined by $T_{c}$. Suppose that $V=\left(0,1,\ldots ,\;N\right)$ is the subset of $E=\left(a,b\right)\left|a,b\in V,\;a\neq b\right|$, where $\left(a,b\right)$ are the neighbors and can also be denoted as a~b. The number of neighbors of each vertex is equal to the number of edges or degree of that vertex. A path of length “r” from vertex *a* to *b* is a series of r+1 vertices that start from *a* and ends at *b* such that the consecutive vertices are adjacent.

For the cluster allocation, the algorithm has to find the main or the final cluster using an optimal route *N*th division; where *N* is equal to the UAVs clusters, which is decided by $F_{c}=\left(b=1,2,\ldots N\right)$. Where “F” is the weighing scale of nodes at each cluster that will reach the final cluster “$F_{c}$” if it satisfies $\overset{N}{\underset{P=1}{⋃}}F_{c}=F$. For more details, the reader is referred to [32]. Built by following [32], the issue of dividing the clustering model is rewritten as:

(1) $$Min\;\sum_{a=1}^{T_{c}}\sum_{b=1}^{N}x_{a,b}d\left(t_{c},c_{P}\right)$$

$x_{a,b}$ is the variable for binary decisions. $x_{a,b}$ is equal to unity if and only if the destination $t_{c}$ belongs to the cluster, whose center is $c_{P}$; otherwise, it will be zero. $c_{P}=\left(b=1,2,\ldots N\right)$ is the center of the cluster and $d\left(t_{c},c_{P}\right)$ is the distance between $t_{c}$ and $c_{P}$.

### 3.3. System Model

Assume *n* agents in a cluster, all agents must be able to share their information according to the following [30,31]:

(2) $$n_{a}=(b|a\sim b)\;\subseteq (1,\;\ldots ,\;n)\backslash (a)$$

The set of agents which are neighbor to agent *a* is $n_{a}$, and the members of the set can strongly link with each other. Now, suppose that there is a predefined radius ℝ, which determines the relationship between the neighbors. Now, the position of the agent *a, (a* = 1, *…*, *n)* in the universal coordinate system is rewritten as $\left(x_{a},y_{a}\right)$, and $V_{a}={\left({\dot{x}}_{a},{\dot{y}}_{a}\right)}^{T}$ is its velocity. The orientation and heading of agent *a* is $\theta_{a}$ and can be written as:

(3) $$\theta_{a}=\tan^{-1}\left({\dot{y}}_{a},{\dot{x}}_{a}\right)$$

Now it is assumed that all the agents inside the cluster are moving with the constant velocity that is V. Moreover, suppose a model of the kinematic agent, which is given as:

(4) $$\left\{\begin{array}{c} {\dot{x}}_{a}=V\times cos(\theta_{a})\\ {\dot{y}}_{a}=V\times cos(\theta_{a})\\ {\dot{\theta }}_{a}=\sigma_{a}\;a=1,2,\;\ldots ,\;n \end{array}\right.$$

Now the main focus is to control the input $\sigma_{a}$, after that, the cluster of autonomous agents is considered to flock when all the agents manage to have the same velocity, and distances between the agents are steady.

**Figure 4:**
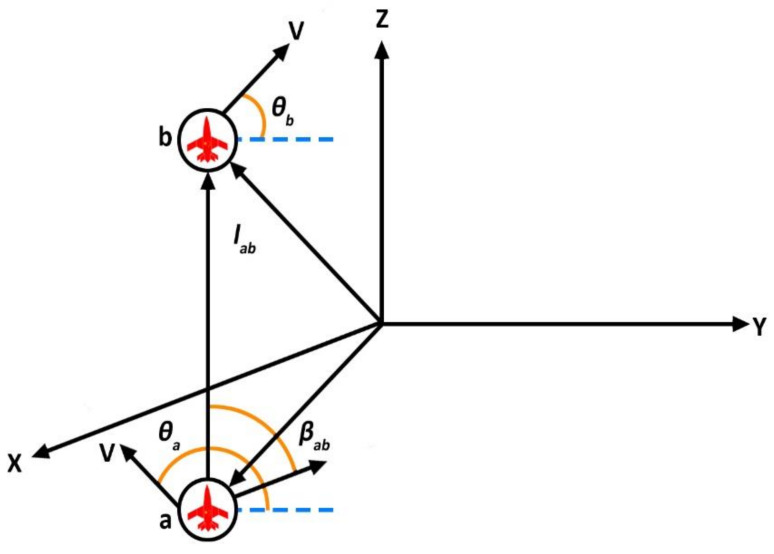
The configuration between two agents in a 3D environment revealing the relative distance and bearing between agent *a* and *b*.

Figure 4 represents the configuration between two agents in a 3D environment inspired by [30]. To adjust the configuration so that it is biologically probable, limit what each agent can do. $\beta_{ab}$ is the bearing associated with the relative angle among agent *a* and agent *b* calculated in the coordinates of agent *a*. To properly demonstrate the concept of sharing, suppose that individual agent *a* can be calculated in terms of ($\beta_{ab}$); the relative bearing in agent-related frame; the change of its bearing rate; and $(\tau_{ab}$) its time to avoid collision concerning any agent *b* in the group of neighbors $n_{a}$. Notice that computation of instant collision does not correspond to the calculated relative distance amongst the agents. Now, plain analysis shows that the bearing and relative length between agents *a* and *b* can be written as:

(5) $$\left\{\begin{array}{c} \beta_{ab}=\tan^{-1}\left(y_{b}-y_{a};\;x_{b}-x_{a}\right)-\theta_{a}+\left(\pi /2\right)\\ l_{ab}^{2}={\left(x_{b}-x_{a}\right)}^{2}+{\left(y_{b}-y_{a}\right)}^{2} \end{array}\right.$$

Now, comparable suppositions can be made for the interaction of agents inside a cluster to extend the model presented above to a 3D space. It is also supposed that all agents are moving with constant velocity [30]. The velocity in term of agent *a* rewritten as:

(6) $$V_{a}={\left[\cos(\theta_{a})\sin\left(\varphi_{a}\right);\;\sin\left(\theta_{a}\right)\sin\left(\varphi_{a}\right);\;\cos\left(\varphi_{a}\right)\right]}^{T}$$

In the above equation, $\theta_{a}$ and $\varphi_{a}$ are the heading and attitude angles, which are written in terms of agent *a.* Now the dynamic equation of the agent is;

(7) $${\dot{V}}_{a}=U_{a\theta }X_{a\theta }+U_{a\varphi }X_{a\varphi }$$

The orthonormal vectors are $X_{a\theta }={\left(-sin\theta_{a};cos\theta_{a};0\right)}^{T}$ and $X_{a\varphi }={\left(cos\theta_{a}cos\varphi_{a};sin\theta_{a}cos\varphi_{a};-sin\varphi_{a}\right)}^{T}$. Now, $U_{a\theta }$ and $U_{a\varphi }$ are the inputs that are written in terms of agent a and are given as;

(8) $$\left\{\begin{array}{c} U_{a\theta }=-\underset{b\in n_{a}}{\sum }\sin(\varphi_{b})\sin\left(\theta_{a}-\theta_{b}\right)\\ U_{a\varphi }=-\underset{b\in n_{a}}{\sum }\sin\left(\varphi_{a}\right)\cos\left(\varphi_{b}\right)-\sin(\varphi_{b})\cos(\varphi_{a})\cos\left(\theta_{a}-\theta_{b}\right) \end{array}\right.$$

The above inputs were developed for the arrangement of velocity vectors of a group of kinematic agents.

## 4. Designed Algorithm

The design approach for this study will be to use the PSO algorithm to find the best agent and then implement Vicsek MAS for synchronization and connectivity between the two different clusters. This strategy is explained in detail below:

### 4.1. Particle Swarm Optimization

PSO is an optimization method inspired by the behavior of a flock of birds or a school of fish. It starts with each particle having a random position and velocity. Then, it continuously updates the position and velocity of each particle until the global best solution is found [33,34]. Particles revise their positions according to the previous value, their individual best values, and the global best value. Their new positions mainly depend on their previous positions and the present velocities [35]. If the current position is better than the earlier positions, it turns into the individual best position for that particle. If the current position of the particle is better than all the particles’ positions, then it is set as the global best value [36,37]. Given the route planning is in a three-dimensional plane and each particle’s waypoint is *S*, so for the *i*th particle, the position vector $P_{i}$ and the velocity vector $V_{i}$ can be given as:

(9) $$\left\{\begin{array}{c} P_{i}={\left[P_{\left(i,1\right)},\ldots ,P_{\left(i,S\right)}\right]}^{T}={\left[{\left(P^{x},P^{y},P^{z}\right)}_{\left(i,1\right)},\ldots ,{\left(P^{x},P^{y},P^{z}\right)}_{\left(i,S\right)}\right]}^{T}\\ V_{i}={\left[V_{\left(i,1\right)},\ldots ,V_{\left(i,S\right)}\right]}^{T}={\left[{\left(V^{x},V^{y},V^{z}\right)}_{\left(i,1\right)},\ldots ,{\left(V^{x},V^{y},V^{z}\right)}_{\left(i,S\right)}\right]}^{T} \end{array}\right..$$

In the above equation, j∈1,…,S; ${\left(P^{x},P^{y},P^{z}\right)}_{\left(i,j\right)}$ and ${\left(V^{x},V^{y},V^{z}\right)}_{\left(i,\;j\right)}$ denote the position and velocity of the *j*th waypoint for the *i*th particle in a 3-dimensional plane. 

Given that *D* denotes the total number of particles, the swarm can be represented as:

(10) $$\left[\left(P_{1},V_{1}\right),\left(P_{2},V_{2}\right),\ldots ,\left(P_{D},V_{D}\right)\right]$$

For a swarm containing *D* particles, there are *D* local best and global best solutions:

(11) $$P_{\left(i,best\right)}={\left[P_{\left(i,1,best\right)},\ldots ,P_{\left(i,S,best\right)}\right]}^{T}={\left[{\left(P^{x},P^{y},P^{z}\right)}_{\left(i,1,best\right)},\ldots ,{\left(P^{x},P^{y},P^{z}\right)}_{\left(i,S,best\right)}\right]}^{T}$$

(12) $$\begin{array}{c} P_{best}^{G}={\left[P_{1,best},\ldots ,P_{S,best}\right]}^{T}\\ \; \end{array}={\left[{\left(P^{x},P^{y},P^{z}\right)}_{\left(1,best\right)},\ldots ,{\left(P^{x},P^{y},P^{z}\right)}_{\left(S,best\right)}\right]}^{T}$$

In the above equation, *i* ∈ 1, …, *D*. The multiple objective PSO function is given as:

(13) $$P_{\left(i,best\right)}\left(t+1\right)=\left\{\begin{array}{c} P_{\left(i,best\right)}\left(t\right);if\;f\left(P_{\left(i,best\right)}\left(t\right)\right)\leq f\left(P_{i}\left(t+1\right)\right)\\ P_{i}\left(t+1\right);if\;f\left(P_{\left(i,best\right)}\left(t\right)\right)>f\left(P_{i}\left(t+1\right)\right) \end{array}\right.$$

(14) $$P_{best}^{G}\in \left(P_{\left(i,1,best\right)},\ldots ,P_{\left(i,S,best\right)}\right)$$

(15) $$f\left(P_{best}^{G}\left(t\right)\right)=\min\left[f\left(P_{best,1}\left(t\right)\right),\ldots ,f\left(P_{best,S}\left(t\right)\right)\right]$$

The position and velocity of the particles in the swarm update according to the following equations:

(16) $$\left\{\begin{array}{c} P_{i,j}\left(t+1\right)=P_{i,j}\left(t\right)+V_{i,j}\left(t+1\right)\\ V_{i,j}\left(t+1\right)=w_{i}\times V_{\left(i,j\right)}\left(t\right)+a_{1}\times r_{1}\times \left(P_{\left(i,j,best\right)}\left(t\right)-P_{\left(i,j\right)}\left(t\right)\right)+a_{2}\times r_{2}\times \left(P_{\left(G,j\right)}^{best}\left(t\right)-P_{\left(i,j\right)}\left(t\right)\right) \end{array}\right.$$

In the above equation, coefficients of acceleration are *a_1_* and *a_2_*, while *r_1_* and *r_2_* represent any number from 0 to 1. Also, *w_i_* represents the inertial weight.

By changing *w_i_*, the performance of the algorithm can be fine-tuned. It can help in stabilizing the individual and global best results of each particle. For a better global outcome, increase *w_i_*, and to enhance the local results, decrease it. The inertial weight is given as:

(17) $$w_{i}=\frac{\left(w_{i}{}_{max}-w_{i}{}_{min}\right)t_{c}}{T_{m}}$$

In the above equation, *t_c_* represents the current instance of the PSO, *T_m_* denotes the highest number of iterations, and $w_{i}{}_{max}$ and $w_{i}{}_{min}$ present the highest and lowest value of *w_i_*.

### 4.2. Vicsek Model

The Vicsek MAS model consists of *n* independent agents traveling with identical speeds through the model. It revises the direction of each agent according to the heading of its neighbor agent. The neighboring agents of agent *a* (1 ≤ *a* ≤ *n*) at any time instance *t* are within a radius *r* centered at agent *a* and are denoted as *N_a_*(*t*),

(18) $$\left\{\begin{array}{c} N_{a}\left(t\right)=\left\{b|d_{ab}\left(t\right)<r\right\}\\ d_{ab}\left(t\right)=\sqrt{{\left(x_{a}\left(t\right)-x_{b}\left(t\right)\right)}^{2}+{\left(y_{a}\left(t\right)-y_{b}\left(t\right)\right)}^{2}} \end{array}\right.$$

In the above equation, the coordinates of agent *a* are (*x_a_*(*t*), *y_a_*(*t*)) at time *t*. The neighboring agent of agent *a* is agent *b*. All the agents in the model travel with the same velocity *v*.

(19) $$\left\{\begin{array}{c} x_{a}\left(t+1\right)=x_{a}\left(t\right)+v\cos\theta_{a}\left(t\right)\\ y_{a}\left(t+1\right)=y_{a}\left(t\right)+v\sin\theta_{a}\left(t\right)\\ \theta_{a}\left(t+1\right)=\arctan\frac{\sum_{b\in N_{a}\left(t\right)}\sin\theta_{b}\left(t\right)}{\sum_{b\in N_{a}\left(t\right)}\cos\theta_{b}\left(t\right)} \end{array}\right.$$

In the above equation, the heading angle of agent *a* is given as *θ_a_*(*t*). The Vicsek MAS is a dynamic model, and this paper uses basic graph theory (discussed in Section 3) to examine this system. The equation for *θ_a_*(*t*) can be rewritten as,

(20) $$\tan\theta_{a}\left(t+1\right)=\sum_{b\in N_{a}\left(t\right)}\frac{\cos\theta_{b}\left(t\right)}{\sum_{k\in N_{a}\left(t\right)}\cos\theta_{k}\left(t\right)}\tan\theta_{a}\left(t\right)$$

(21) $$\tan\theta \left(t+1\right)=A\left(t\right)\tan\theta \left(t\right)$$

In the above equation, $\tan\theta \left(t\right)≜{\left(\tan\theta_{1}\left(t\right),\;\ldots ,\tan\theta_{N}\left(t\right)\right)}^{\tau }$, $A\left(t\right)$ is the average weighted matrix of the graph ${\hat{G}}_{t}$ and is given as;

(22) $${\hat{a}}_{ab}\left(t\right)=\left\{\begin{array}{cc} \frac{\cos\theta_{b}\left(t\right)}{\sum_{k\in N_{a}\left(t\right)}\cos\theta_{k}\left(t\right)} & if\;\left(a,\;b\right)\in E_{t}\\ 0, & otherwise \end{array}\right.$$

To analyze the synchronizing behavior of the Vicsek model, the linear version of the *θ_a_*(*t*) can be written as:

(23) $$\theta_{a}\left(t+1\right)=\frac{1}{n_{a}\left(t\right)}\sum_{b\in N_{a}\left(t\right)}\theta_{b}\left(t\right)$$

In the above equation, $N_{a}\left(t\right)$ contains $n_{a}\left(t\right)$ elements. Likewise, Equation (22) can be rewritten as;

(24) $$\tan\theta \left(t+1\right)=\tilde{A}\left(t\right)\theta \left(t\right)$$

In the above equation, $\theta \left(t\right)≜{\left(\theta_{1}\left(t\right),\;\ldots ,\theta_{N}\left(t\right)\right)}^{\tau }$, and the elements of the matrix $\tilde{A}\left(t\right)$ can be given as

(25) $${\tilde{a}}_{ab}\left(t\right)=\left\{\begin{array}{ll} \frac{1}{n_{a}\left(t\right)}, & if\;\left(a,b\right)\in E_{t}\\ 0, & otherwise \end{array}\right.$$

### 4.3. Synchronization and Connectivity

To better understand the synchronization and connectivity of the Vicsek model, first, this study must clearly describe synchronization. The above-mentioned Vicsek model is said to synchronize when the heading angles of all the agents satisfy the following equation:

(26) $$\underset{t\to \infty }{\lim}\theta_{a}\left(t\right)=\theta ,\;a=1,\;\ldots ,\;N$$

In the above equation, *θ* depends on the initial values $\left\{\theta_{a}\left(0\right),\;x_{a}\left(0\right),\;y_{a}\left(0\right),\;a=1,\ldots ,N\right\}$ and the parameters of *r* and *v*. The propositions given below will establish the synchronization for the Vicsek model and its linearized version.

Considering the Vicsek model of Equation (20), suppose that the starting neighbor graph ${\hat{G}}_{0}=\{V,\;E_{0}\}$ is connected and $\left\{\theta_{a}\left(0\right)\in \left(-\frac{\pi }{2},\frac{\pi }{2}\right),\;a=1,\;\ldots \;,\;N\right\}$, therefore, the Vicsek model will synchronize if it satisfies the condition:

(27) $$\left\{\begin{array}{c} v\leq \frac{d}{{△}_{0}}{\left(\frac{\cos\bar{\theta }}{N}\right)}^{N}\\ \bar{\theta }=\underset{i}{\max}\left|\theta_{a}\left(0\right)\right|\\ d=r-\underset{a,b\in E_{0}}{\max}d_{ab}\left(0\right)\\ {△}_{0}=\underset{a,b}{\max}\left\{\tan\theta_{a}\left(0\right)-\tan\theta_{b}\left(0\right)\right\} \end{array}\right.$$

In the above equation, *N* represents the number of total agents. Considering the linear version of the model in (20) and (24), let the starting graph ${\hat{G}}_{0}$ be connected and *θ_a_*(0) ∈ [0, 2π). Therefore, the Vicsek model will synchronize if it satisfies the condition:

(28) $$v\leq \frac{d{\left(\frac{1}{N}\right)}^{N}}{2\pi }$$

### 4.4. Dynamic Leader Selection

The formation configuration sometimes undergoes variations in systems with multiple agents because of communication failures among agents. To model a random communication failure, each link (*a*,*b*) ∈ *E* individually fails with *p* probability. Let the graph topology for this communication failure model be $\hat{G}$ and $E_{\hat{G}}\;\left(t_{conv}\right)$ be the estimated convergence time. While $E_{\hat{G}}()$ represents the expectation of the argument over the group of network formations, denoted by $\hat{G}$. Decreasing $E_{\hat{G}}\;\left(t_{conv}\right)$ will maximize the convergence rate. Therefore, the formula to select a leader *k* for maximizing the convergence rate is,

(29) $$\underset{Y}{max}E_{\hat{G}}\left(\min_{x\left(0\right)}x{\left(0\right)}^{T}\left(Y\hat{L}+\hat{L}Y\right)x\left(0\right)\right)$$

Such that it satisfies the following conditions,

(30) $$\left\{\begin{array}{c} tr\left(Y\right)\geq n-k\\ Y_{aa}\in \left\{0,1\right\}\forall_{a}\neq V\\ Y_{ab}=0\forall_{a}\neq b \end{array}\right.$$

The objective function is consistent with the expected convergence rate over the potential network formations. As $\min_{x\left(0\right)}x{\left(0\right)}^{T}\left(Y\hat{L}+\hat{L}Y\right)x\left(0\right)$ is a convex function of *Y*, which is the MAS convergence rate, the objective function of (30) is also convex because it is the weighted sum of convex functions.

### 4.5. B-Spline Path Smoothing

The path planned by the hybrid algorithm mostly consists of a series of line segments. To make sure that the path generated is smooth and flightworthy, the B-spline curve strategy is used. The B-spline curve is an improvement of the Bezier curve method and has the advantages of preserving convexity and geometrical invariability.

Mathematically, the B-spline curve can be given as [38];

(31) $$P\left(u\right)=\sum_{a=0}^{n}d_{a}N_{a,b}\left(u\right)$$

In the above equation, $d_{a}\left(i=0,1,\ldots ,n\right)$ are the controlling points, and $N_{a,b}\left(u\right)$ are the normalized b-order B-spline functions and are defined with the help of the Cox-de Boor recursion method as;

(32) $$\left\{\begin{array}{l} N_{a,b}\left(u\right)=\left\{\begin{array}{l} 1,\;if\;u_{a}\leq u\leq u_{a+1}\\ 0,\;otherwise \end{array}\right.\\ N_{a,b}\left(u\right)=\frac{u-u_{a}}{u_{a+b}-u_{a}}N_{a,b-1}\left(u\right)+\frac{u_{a+b+1}-u}{u_{a+b+1}-u_{a+1}}N_{a+1,b+1}\left(u\right)\\ define\frac{0}{0}=0 \end{array}\right.$$

The parametric knots $\left\{u_{0}\leq u_{1}\leq \ldots \leq u_{n+b}\right\}$ determine the basic functions of the B-spline curve. The B-spline curve is not affected by just moving a single control point, as opposed to the Bezier curve. Another advantage over the Bezier curve is that increasing the control points will not increase the degree of polynomials.

## 5. Flowchart and Algorithm

**Figure 5:**
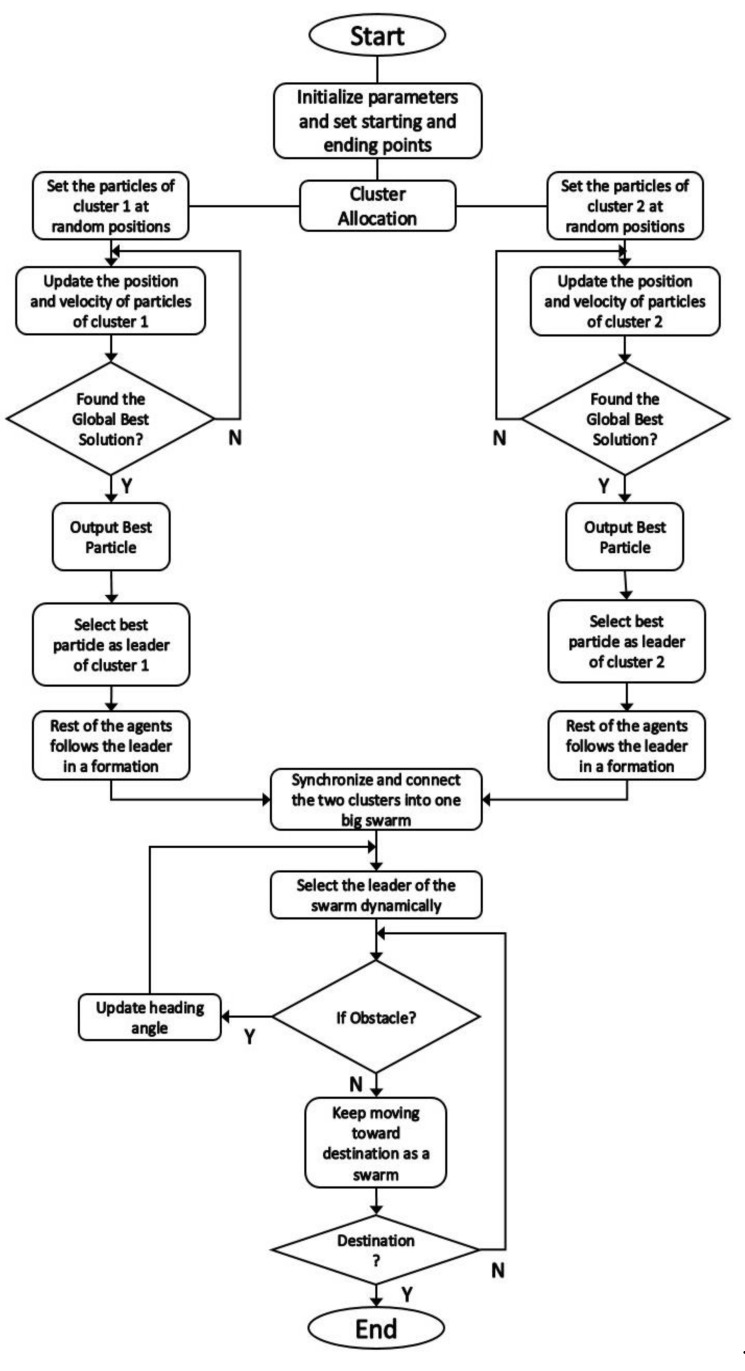
Flowchart of the Proposed Method.

This section details the flowchart and the algorithm of the proposed method. Figure 5 presents the flowchart of the designed strategy. As it is clear from the flowchart, the algorithm starts by initializing the parameters and setting the starting and ending points for the mission. Afterward, the algorithm performs the cluster allocation. Then, it sets the particles of cluster 1 and 2 at random positions and starts updating their position and velocity until it finds the global best solution. Later, MAS designates that particle found in the global best solution as the leader of that cluster, and the rest of the particles start to follow the leader as agents in a formation.

Moreover, the Vicsek model ensures synchronization and connectivity within each cluster and the larger swarm as well. Finally, the strategy uses dynamic leader selection to select the leader of the swarm and determines if there is any obstacle in the path of the swarm. If there is an obstacle, the algorithm updates the heading angles of the agents and selects a different leader for the swarm dynamically according to the mission requirement. The strategy repeats this process until the destination is found.

The Algorithm 1 of the proposed method is detailed below:
**Algorithm 1** The pseudo code 
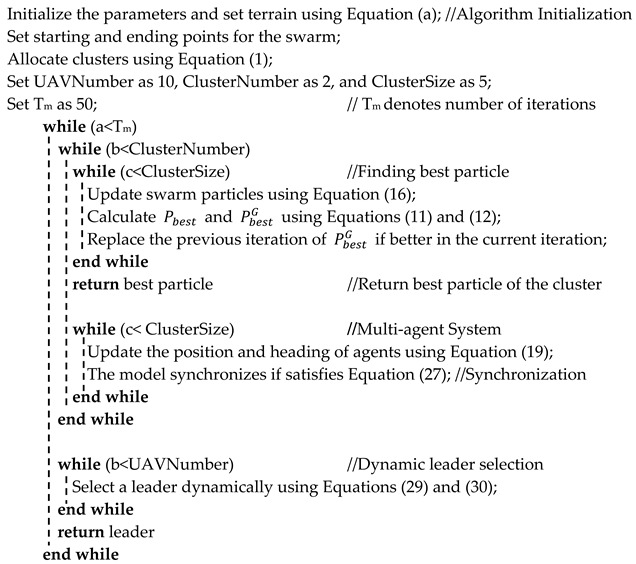


## 6. Simulations

This section proves the efficacy of the proposed hybrid algorithm using simulations. The mission area is 30 km long, 35 km wide, and the altitude is 3 km. First, this study compares the performance of the proposed method with the NSGA-II genetic algorithm. Then, the study will implement the designed algorithm in two different scenarios. The first scenario contains two clusters of five UAVs each at random positions. The task is to bring these UAVs into the desired formation using PSO and MAS. The second task concerns joining the two clusters to form one big swarm. The synchronization and connectivity between the UAVs are ensured by the Vicsek MAS model.

### 6.1. Comparison with NSGA-II

**Figure 6:**
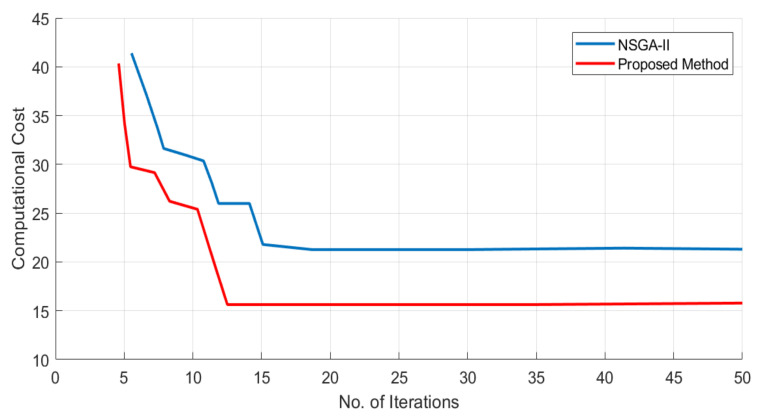
Performance comparison between the proposed method and the NSGA-II; the *y*-axis represents the computational cost, and the *x*-axis shows the number of iterations.

To test the efficiency of the designed strategy, this paper first compares it with the genetic algorithm NSGA-II. Figure 6 shows the comparison between the computational cost of the NSGA-II and the proposed method. As it is clear from the figure, the proposed method requires less computational power as compared to NSGA-II. Also, the proposed method takes fewer iterations to find the optimal solution.

**Figure 7:**
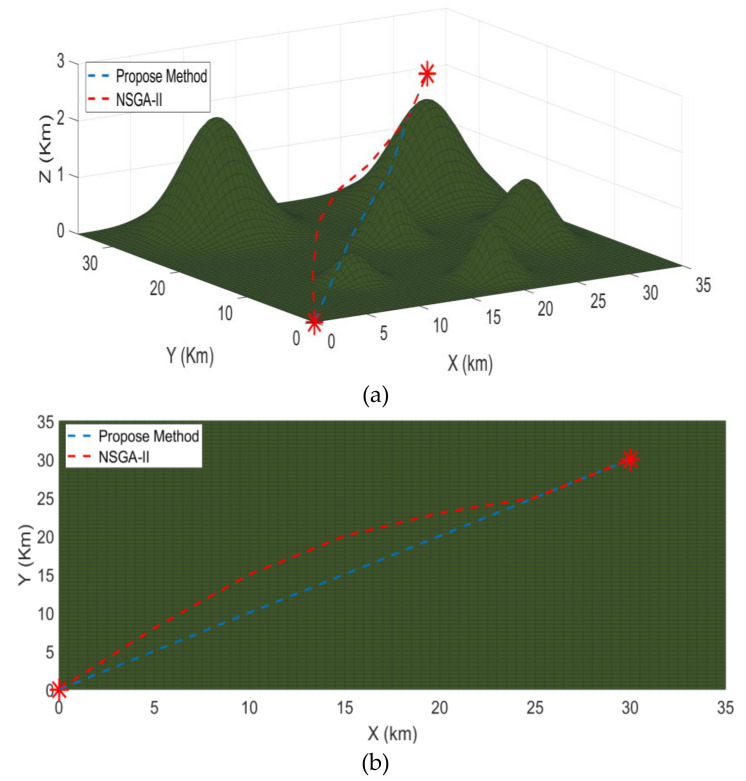

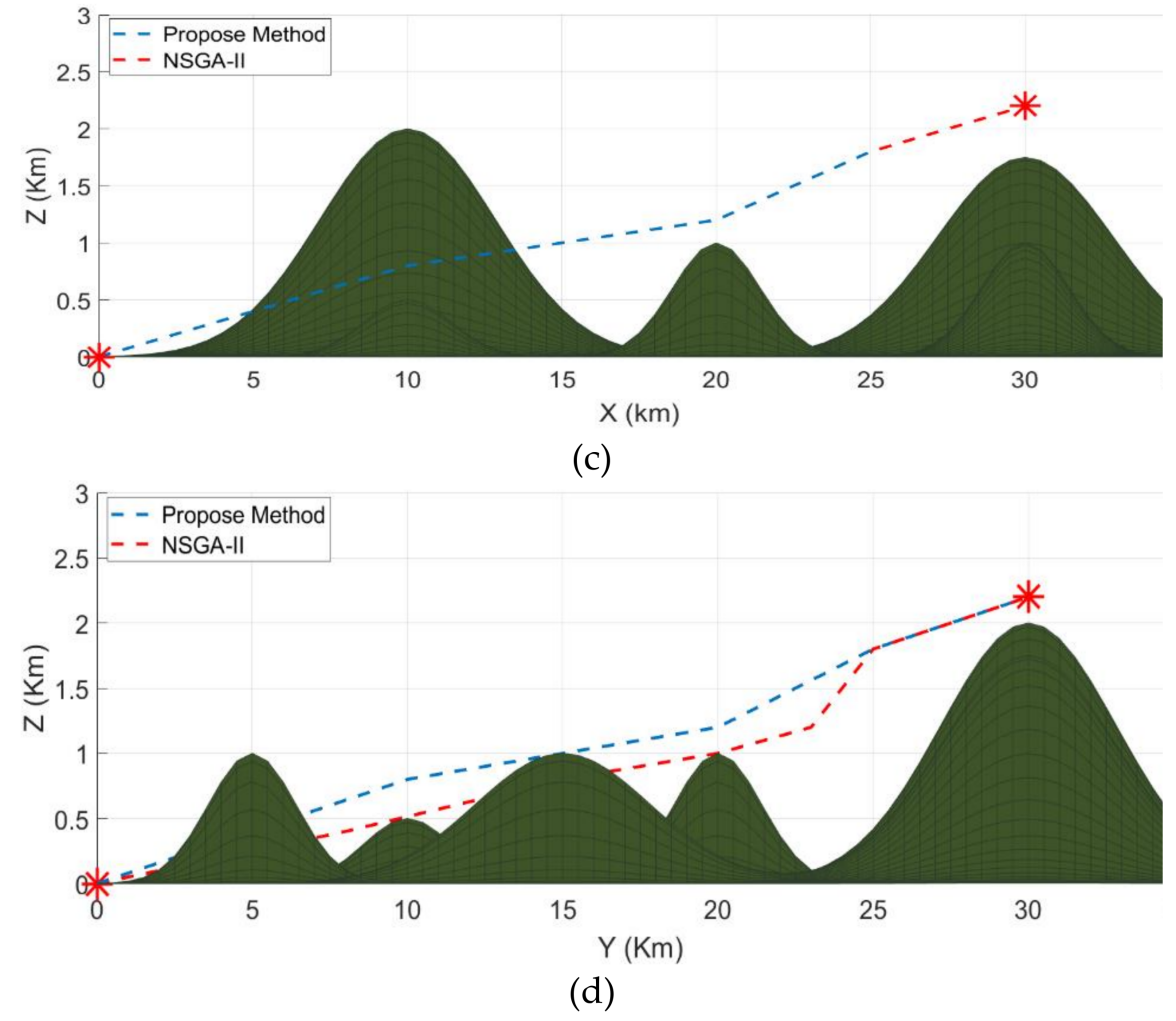
Comparison between NSGA-II and the proposed method; (**a**) shows the 3D version while (**b**–**d**) represent the *XY*, *XZ*, and *YZ* axis versions, respectively. The legend is the same for all simulations.

Figure 7 presents the comparison between the two algorithms from a different perspective. Figure 7a shows the 3D version while Figure 7b–d represent the *XY*, *XZ*, and *YZ* axis versions, respectively. It is clear from the figure that the proposed method is more optimal than the NSGA-II. It takes less time and distance to arrive at the destination while still avoiding the obstacles. It is straighter and takes less turns, which help in remaining stable during the flight.

### 6.2. Problem Case 1

**Figure 8:**
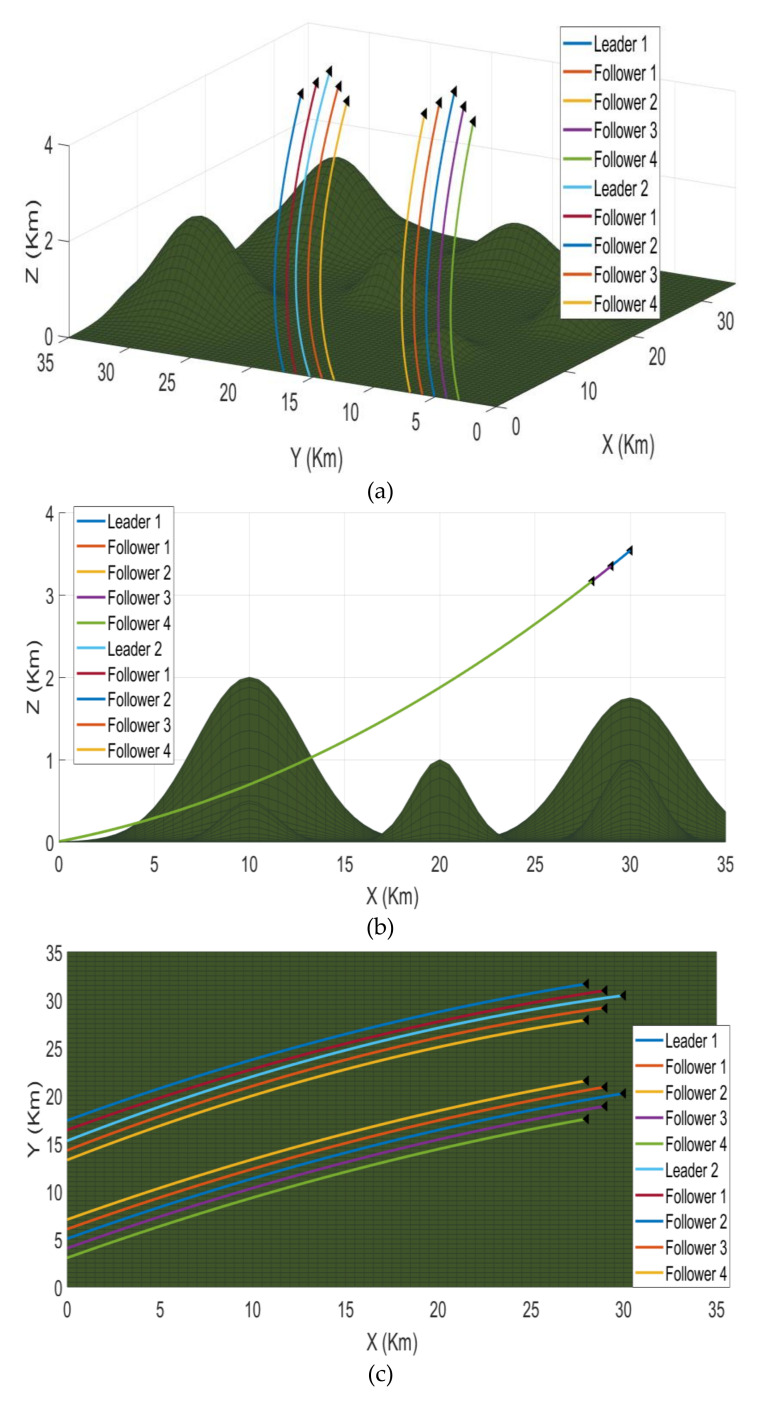

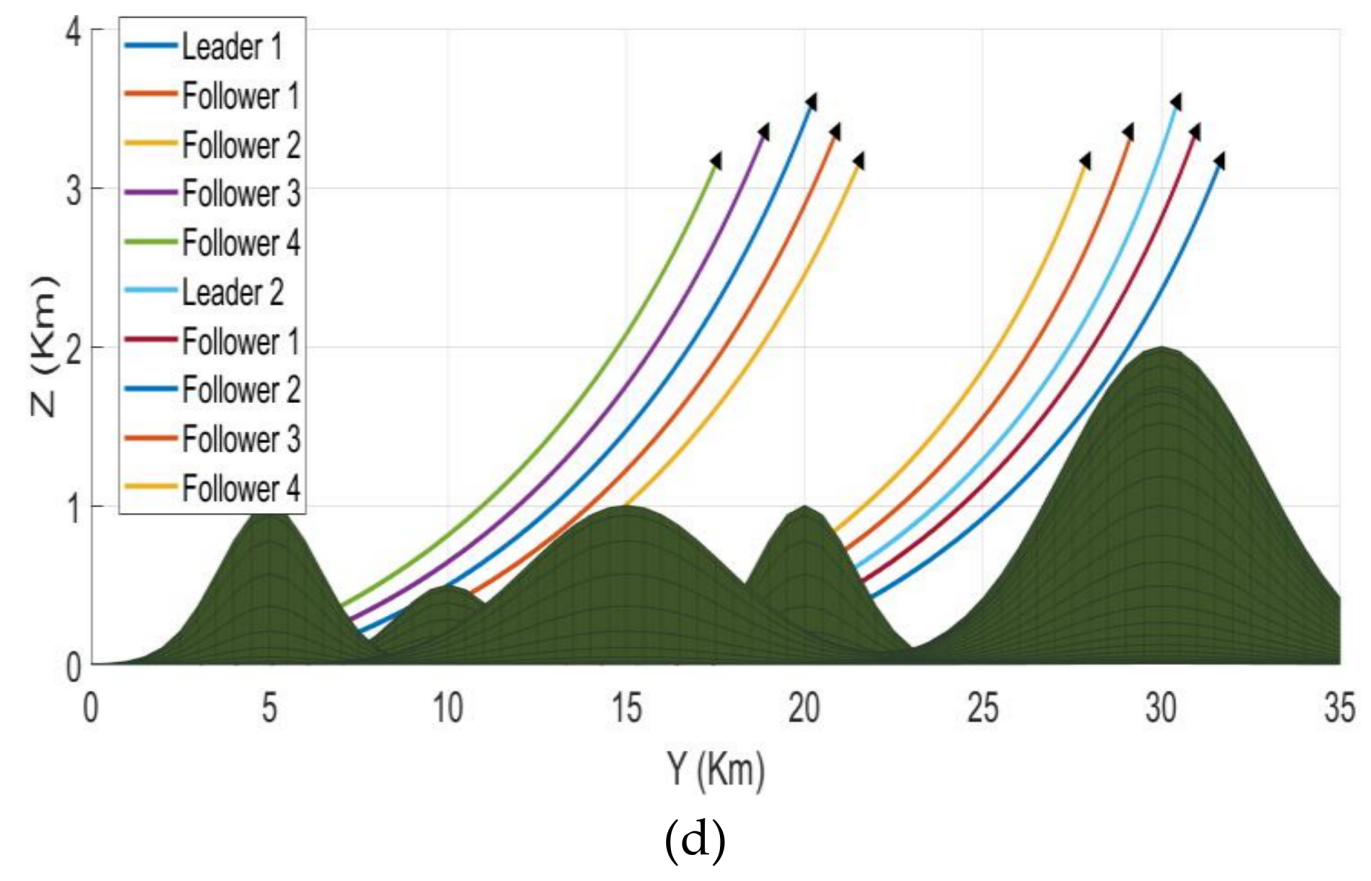
The first mission scenario containing two clusters with five UAVs each reaching a leader-follower formation; (**a**) shows the 3D version while (**b**–**d**) represent the *XZ*, *XY*, and *YZ* axis versions, respectively. The legend is the same for all simulations.

The first scenario deals with organizing the two clusters into formations and then maintaining that formation for the second scenario. In the first scenario, 10 fixed-wing UAVs are divided into two clusters and each cluster has a leader and four followers. It means five UAVs in each cluster work collaboratively to perform a task. The terrain model contains different obstacles like mountains. The aerial vehicles have a challenging time avoiding the hazardous peaks, maintaining their strategy, and reaching the designated area safely. Hence, it is necessary to maintain the desired synchronization and connectivity with their neighbor agents. Figure 8 presents different perspectives of the first scenario. The Figure 8a shows the 3D version while Figure 8b–d represent the *XZ, XY,* and *YZ* axis versions, respectively. First, PSO finds the best particle in each cluster, and then the MAS designates that particle as the leader of that cluster. Afterward, the rest of the UAVs start to follow in a leader-follower formation. As it is clear in the simulation, the two clusters are now in the leader-follower formation with two leaders each having four followers. The two clusters maintain their formation even through the mountainous terrain and narrow spaces, as evident from the top-right simulation. The proposed method performed the task perfectly and the two clusters are now ready to be synchronized into one big cluster in the next scenario.

### 6.3. Problem Case 2

**Figure 9:**
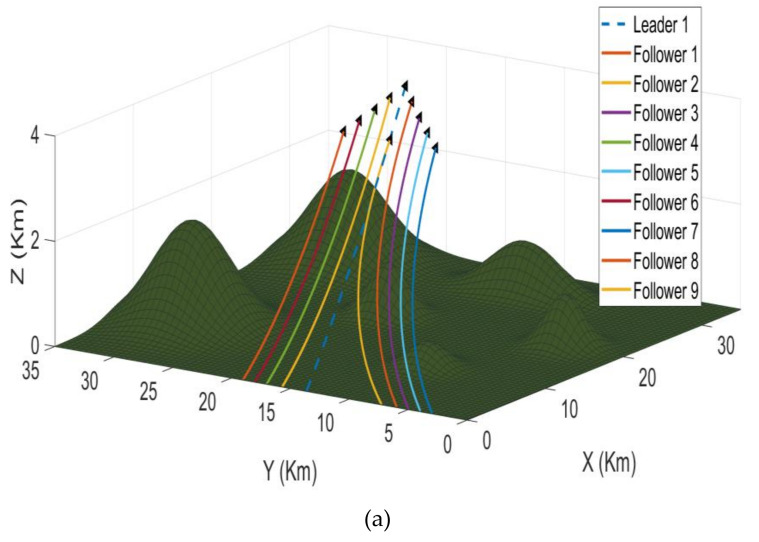

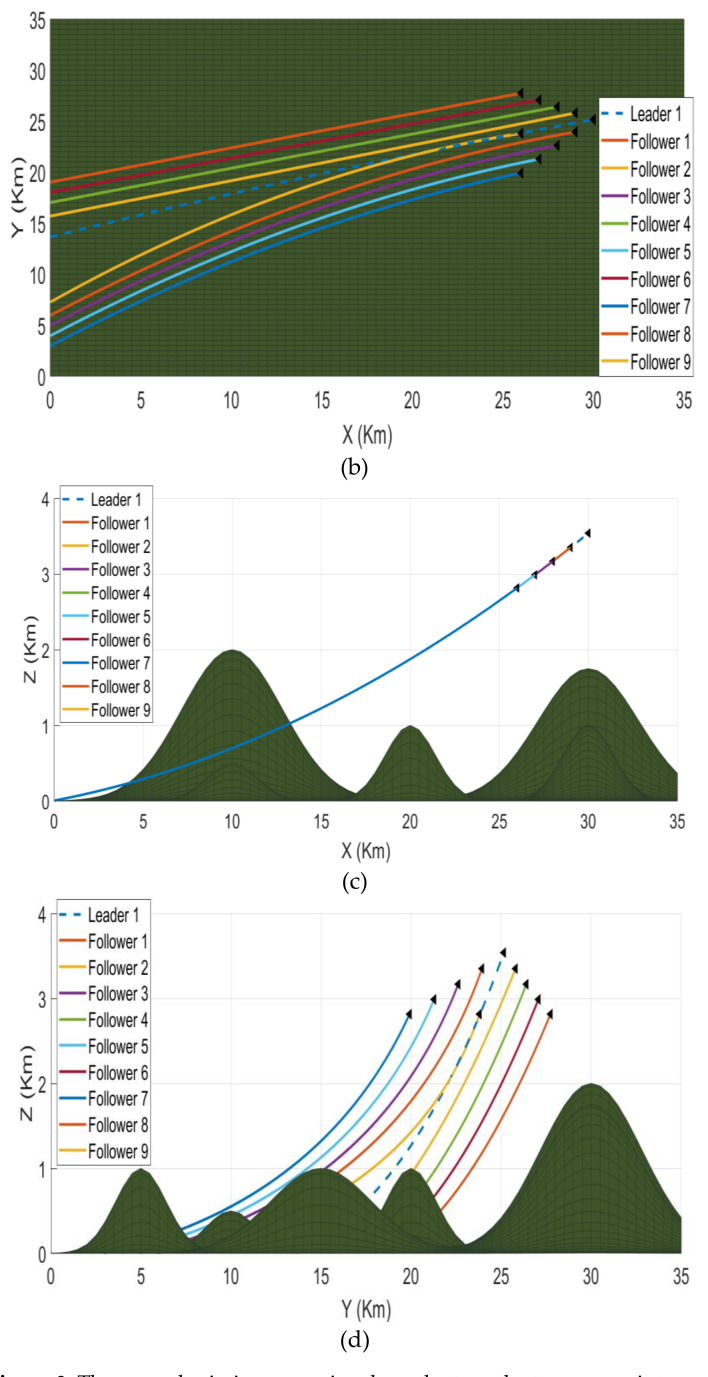
The second mission scenario where the two clusters merge into one big swarm; (**a**) shows the 3D version while (**b**–**d**) represent the *XY*, *XZ*, and *YZ* axis versions, respectively. The legend is the same for all simulations.

The second scenario starts where the first scenario ended. The second scenario starts after successfully maintaining the formation of both the clusters and fulfilling their separate mission tasks in the first scenario. Afterward, both the clusters join in a common cluster called a swarm. At that instant of time, perfect coordination or communication between both the clusters is required to choose which leader will lead the formation of a swarm now. To help achieve the above objective, the algorithm will dynamically select the leader. Figure 9 presents different perspectives of the second scenario in simulation. The Figure 9a shows the 3D version while Figure 9b–d represent the *XY*, *XZ*, and *YZ* axis versions, respectively. As it is clear, the algorithm has transformed the two clusters into one swarm with the leader being selected dynamically.

## 7. Conclusions

This study proposed a collective motion and self-organization control of a swarm of 10 UAVs, which were divided into two clusters. This article designed a hybrid meta-heuristic algorithm by merging the particle swarm optimization (PSO) with the multi-agent system (MAS). PSO provided the best agents of a cluster. Afterward, the MAS assigned the best agent as the leader of the cluster. Moreover, the leader found the optimal path for each cluster. Initially, each cluster had agents at random positions. Later, the clusters were organized into a formation by implementing PSO with the MAS model. This helped coordinate the agents inside the cluster. However, for the connectivity and synchronization of the common cluster, this study used the Vicsek model along with dynamic leader selection ability. The flying formations of each cluster and the swarm were created by the B-spline curve based on simple waypoint-defined graph theory. This paper compared the designed algorithm with the NSGA-II to show that the proposed model has better convergence and durability both inside the clusters and inside the greater swarm. Lastly, the simulations showed that the algorithm is effective and completed both the scenarios according to the mission requirements.

The proposed algorithm can be applied to any number of clusters with any number of UAVs. However, this study focused on a specific scenario of 10 UAVs with two clusters. For future plans, the authors plan to apply the proposed method to more clusters and with more UAVs than 10. The authors also intend to compare the efficiency of the proposed method with other state-of-the-art algorithms in the near future. The authors also plan to implement the proposed algorithm on hardware and compare the experimental results with the simulation results.

## Data Availability

All the data used to support the findings of this study are included within the article.
